# The manifestation of participation within a co‐design process involving patients, significant others and health‐care professionals

**DOI:** 10.1111/hex.13233

**Published:** 2021-03-17

**Authors:** Sebastian Lindblom, Maria Flink, Marie Elf, Ann Charlotte Laska, Lena von Koch, Charlotte Ytterberg

**Affiliations:** ^1^ Department of Neurobiology, Care Sciences and Society Karolinska Institutet Huddinge Sweden; ^2^ Karolinska University Hospital Stockholm Sweden; ^3^ School of Education, Health and Social Studies Dalarna University Falun Sweden; ^4^ Department of Clinical Sciences Danderyd Hospital Karolinska Institutet Stockholm Sweden

**Keywords:** design thinking, health services research, involvement, participatory design, patient participation, qualitative research, rehabilitation, stakeholder participation, stroke, user involvement

## Abstract

**Background:**

Despite intentions to increase user participation in the development of health services, the concept of participation and how it unfolds within studies with a participatory design has rarely been addressed.

**Objective:**

The aim of this study was to describe how user participation manifests itself within a co‐design process involving patients, significant others and health‐care professionals, including potential enablers or barriers.

**Methods:**

This study was conducted in the context of a co‐design process of a new person‐centred transition from a hospital to continued rehabilitation in the home involving three patients with stroke, one significant other and 11 professionals. Data were collected by observations during the workshops, semi‐structured interviews and questionnaires.

**Results:**

Four categories: ‘Composition of individuals for an adaptive climate’; ‘The balancing of roles and power’; ‘Different perspectives as common ground for a shared understanding’; and ‘Facilitating an unpredictable and ever‐adaptive process’, with all together nine subcategories, resulted from the analysis. Participation varied between individuals, groups and steps within the process, and on the topic of discussions and the motivation to contribute.

**Discussion/Conclusion:**

Participation is not something that is realized by only applying participatory design methodology. Participation manifests itself through the interaction of the participants and their skills to handle different perspectives, roles and assignments. Participation is enabled by individual, group and facilitating aspects. Co‐design processes should allow for varying levels of participation among the participants and throughout the process.

**Patient or public contribution:**

Patients, significant others and health‐care professionals participated as co‐designers of a care transition model between hospital and home.

## 1 INTRODUCTION

User participation aims at the higher rungs on Arnstein's ladder of citizen participation, that is the redistribution of power by involving those who otherwise might be the subject of research or recipients of care to be deliberately included.^1^ This is recognized as a key component to enhance quality in health and social care.^2^ User participation in health care has been described as a continuum spanning from consultation to partnership and shared leadership on the levels of direct care and organizational design, and in policymaking.^3^, ^4^

On the level of the direct care, user participation has been linked to person‐centred health care with active, informed and empowered patients where health care is provided in a patient‐professional partnership, as opposed to a paternalistic health care where patients are seen as passive recipients of care.^5^, ^6^, ^7^ On the level of organizational design, there has been an increase in the use of participatory study designs, intending to involve relevant users, for example patients, their families and health‐care professionals, to co‐design improvements and develop health services. Such an approach has been suggested to result in health‐care services that better meet the actual needs and expectations of the users and are more likely to be implemented in practice.^8^, ^9^, ^10^ However, it has been shown that these joint processes are complex^11^, ^12^, ^13^ and the empirical evidence is inconclusive.^14^, ^15^, ^16^

Previous research on co‐design has highlighted the risk of unequal power relationships, difficulties making voices heard, barriers to contribute in a meaningful way and a tokenistic approach to involvement.^17^, ^18^, ^19^, ^20^, ^21^ In co‐design processes involving patients and professionals, there may be difficulties to achieve equal power relationships due to inherent hierarchical structures.^22^ Further, in processes involving patients with limited cognitive and communicative skills,^4^ such as after a stroke, it may be even more challenging to achieve equal power relationships. Calls have been made to critically explore participation in health‐care design^23^ and how ‘reconfigurations of power relations’ can be achieved within co‐design processes.^24^ Consequently, research with a specific focus on user participation in the co‐design process and the enablers and barriers to user participation is needed. In this study, we define user participation as participants’ actions and behaviours in the development of a new health service during a co‐design process at the level of organizational design.

Therefore, the aim of this study was to describe how user participation manifests itself within a co‐design process involving patients, significant others and health‐care professionals, including potential enablers or barriers.

## 2 METHODS

We conducted a co‐design process, intending to develop a new care transition process between the hospital and the home with continued rehabilitation in the home environment for people with stroke. The co‐design process was conducted with design thinking, a human‐centred approach and user‐driven development processes that co‐create solutions to problems in collaboration between users.^25^, ^26^ Design thinking is both a process/methodology and an approach. The co‐design process, conducted in the Stockholm region, consisted of five half‐day workshops, held at Openlab, a designlab and challenge‐driven innovation community, starting in November 2019 and ending in January 2020.

Ethical approval was obtained from the Swedish Ethical Review Authority.

### 2.1 Participants

Three people with stroke, one significant other and ten health‐care professionals took part as seen in Tables 1 and 2. To enable participation, the employers of the participants (patients, significant others and professionals) were offered reimbursement for the time the participants were absent from work. The workshops were moderated by a facilitator trained in design thinking. The term patient will be used throughout the paper. However, we do acknowledge that the participants, who had had a stroke and took part within this study, were no longer, by definition, patients.

**TABLE 1:** Characteristics of the patients and significant other

Participant	Sex	Age	Years since stroke	Working	SIS recovery^a^	Number of workshops attended	Interview/questionnaire
Patient 1	Female	57	6	Yes	95	5/5	Interview
Patient 2	Male	92	1.5	Retired	90	5/5	Interview
Patient 3	Female	74	<1	Retired	90	4/5	Interview
Significant other	Female	59	2^b^	Yes		5/5	Interview

^a^ SIS recovery = Stroke Impact Scale self‐rated recovery on a visual analogue scale from 0 to 100, where 0 indicates ‘not recovered at all’ and 100 indicates fully recovered after stroke.^49^

^b^ Years since the stroke onset of the husband.

**TABLE 2:** Characteristics of health‐care professionals (H1‐H10) and facilitator (F1)

Participant	Profession	Number of workshops attended	Interview/questionnaire
H1	Occupational therapist	5/5	Interview
H2	Registered nurse	5/5	Questionnaire
H3	Speech and language therapist	5/5	Questionnaire
H4	Physician	4/5	Questionnaire^a^
H5	Physiotherapist	4/5	Interview
H6	Speech and language therapist	2/5	Questionnaire
H7	Physiotherapist	4/5	Questionnaire
H8	Physiotherapist	3/5	Questionnaire
H9	Physiotherapist	4/5	Questionnaire
H10	Occupational therapist	5/5	Interview
F1	Facilitator	5/5	Interview

^a^ Did not respond to questionnaire.

#### 2.1.1 Patients and significant others

Patients and significant others were recruited through announcements in the patient organizations such as the Swedish Stroke Association and Neuro Sweden. The criteria for inclusion were people who (a) had had a stroke and had experienced the care transition from a hospital to continued rehabilitation in the home within the Stockholm region; or (b) were a significant other to a person meeting the criteria in a; (c) were available to participate in five workshops; and (d) were able to communicate in Swedish.

Among patients, we sought a variation in age, sex and years since diagnosis. In total, 10 patients and one significant other reported their interest in participating in the study. Of these, three patients met the inclusion criteria. Due to the low number of significant others reporting their interest to participate, contacts were made with significant others from a previous study on experiences of care transitions.^27^ Out of three significant others approached in this way, two declined and one agreed to participate. Oral and written information about the study was provided. From those who agreed to participate, a written informed consent was obtained before enrolment in the study.

#### 2.1.2 Health‐care professionals

Eligible to participate were health‐care professionals who were involved in the care transition of people with stroke from a hospital to continued rehabilitation in the home environment. Therefore, an email was sent to the managers of acute stroke units and geriatric stroke units at one county hospital in the Stockholm region, and the managers of two corresponding multidisciplinary rehabilitation teams in primary care, with information about the study and an invitation to participate. From the hospital, five health‐care professionals, including one physician, one physiotherapist, one registered nurse, one speech and language therapist and one occupational therapist, were included. From the two multidisciplinary rehabilitation teams, three physiotherapists, one occupational therapist and one speech and language therapist were included. All health‐care professionals were women with a mean age of 44 (29‐55). The mean time working within their profession was 17 years (4‐30), and the mean time working at the current workplace was 8 years (2‐20).

### 2.2 Co‐design process

The overall focus of the co‐design process was to develop solutions (prototype) to improve the experience of care transitions for patients and significant others from the hospital to continued rehabilitation in the home environment. At the start of the co‐design process, the facilitator emphasized that the experiences of the care transition process of the patients and significant others were in focus. Co‐design activities such as patient narratives, patient journeys, interviews with patients and the significant other were conducted to give precedence to the needs of patients and significant other. The co‐design process comprised five half‐day workshops, held in Swedish, with different elements and modules and breaks in between for food and drinks.^26^, ^28^ The participants were divided into three groups, the same across all five workshops, in order to facilitate discussion and creative collaboration. The groups consisted of one patient per group, and one group also included the significant other. All groups included three professionals of different professions, from both hospital and multidisciplinary rehabilitation teams. The researchers composed the groups based on both personal acquaintances with the participants and the experiences of the care transitions as a patient, significant other or professional.

**FIGURE 1:**
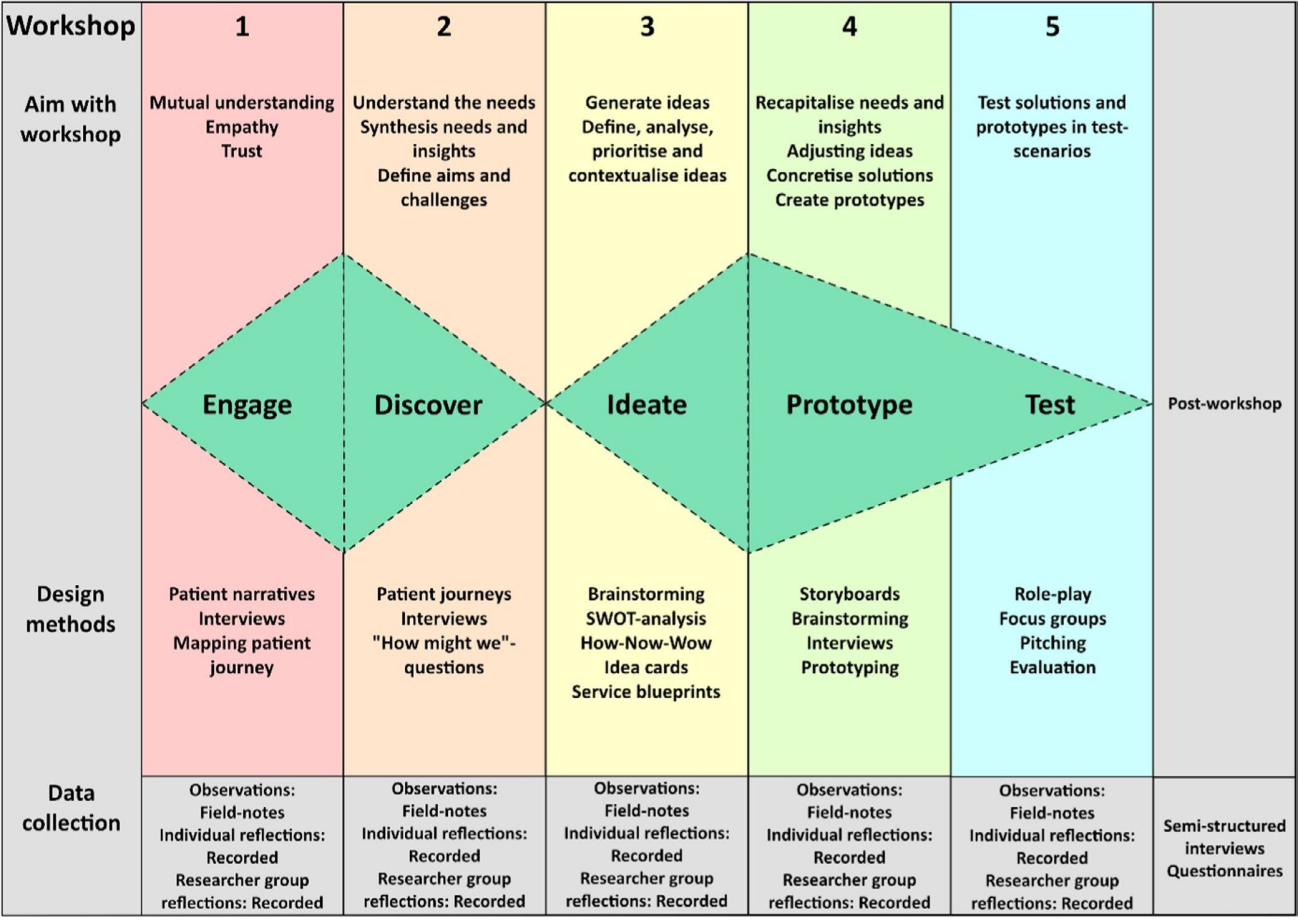
Visualization of the co‐design process including aims, design methods and data collection of each workshop^28^

The co‐design process followed the foundation of the double‐diamond model^28^ and used varying design methods to meet the aim of the specific workshop as seen in Figure 1. Before and in between the workshops, the researchers collaborated with the facilitator in an iterative process of planning and structuring the workshop process.

### 2.3 Data collection

Data were collected by observation, taking field notes and recording post‐observation reflections. After the last workshop, the participants were asked to partake in either a semi‐structured interview or fill out a questionnaire with open‐ended questions about their experience of the co‐design study. The data collection was conducted by three PhDs (MF, CY and LvK) well experienced in qualitative research, and one doctoral student (SL).

#### 2.3.1 Observations

Observations of the co‐design workshops were conducted by three researchers per workshop. The researchers observed one group each and took field notes. The observations were based on the aim of the study. The field notes were transcribed verbatim and were on average 1762 words per workshop.

#### 2.3.2 Reflections

After each workshop, the researchers and facilitator recorded an individual verbal reflection regarding their experiences from observing the workshop. Individual reflections lasted between 2 and 7 minutes, with a mean of 4:14 minutes. After the individual reflection, the researchers held a recorded group reflection. The group reflection lasted between 15 and 37 minutes, with a mean of 26:07 minutes. The reflections were transcribed verbatim.

#### 2.3.3 Semi‐structured interviews

After the completion of all workshops, individual interviews were conducted with the patients, the significant other, one health‐care professional from each co‐design group with variations in the workplace and profession, and the facilitator. The interview guide is presented in Appendix 1. The interviews lasted between 24 and 46 minutes, with a mean of 32:19, and were recorded and transcribed verbatim. As one of the interviewees did not want to be audio‐recorded, notes were taken, and the interview lasted for 50 minutes.

#### 2.3.4 Questionnaires

Those health‐care professionals who were not interviewed were invited to fill in an electronic questionnaire with open‐ended questions. The questionnaire contained the same questions as the semi‐structured interviews. The answers were on average 32 words per question.

### 2.4 Data analysis

Since the aim was to describe how user participation manifests itself within a co‐design process including potential enablers or barriers, qualitative content analysis^29^ was chosen as the data analysis method. The transcribed data from field notes, reflections, interviews and questionnaires were analysed with inductive qualitative content analysis. Each source of data was initially analysed separately with manifest and latent content analyses according to the steps presented in Table 3, in accordance with Graneheim and Lundman.^29^ SL created meaning units, condensed meaning units and coded the data in close collaboration and on‐going discussion with MF and CY. The grouping of codes and creation of subcategories were done by continuous discussions and collaboration between SL, MF and CY. After steps 1‐5, the subcategories from different sources of data were merged and compared by SL, MF, CY and ME to find similarities and differences in data in order to form categories. The subcategories and categories were discussed and refined by all researchers. All researchers discussed and agreed on the final categories. The group of researchers who conducted the analysis represented clinical and scientific expertise in stroke rehabilitation, transitional care and health services research. To strengthen trustworthiness, data were analysed both separately by SL and in discussion within the group. In these discussions, the group critically assessed and reflected upon their prior understanding.

**TABLE 3:** The steps of the analyses

Step 1	The transcribed unit of analysis was read through to get an overview of the content.
Step 2	The unit of analysis was divided into meaning units comprising words, sentences or paragraphs related to each other.
Step 3	The meaning units were condensed without interpretation through the shortening of the text without removing the core meaning of the unit.
Step 4	Each meaning unit was labelled with a code through the interpretation of the underlying meaning.
Step 5	Codes of similar meaning were clustered into subcategories. The initial subcategories were reduced through a back and forward comparison of codes and groups to capture the underlying meaning.
Step 6	Subcategories from the different sources of data were analysed by their latent content and compared in order to form categories based on the underlying meaning of the subcategories.

## 3 RESULTS

**FIGURE 2:**
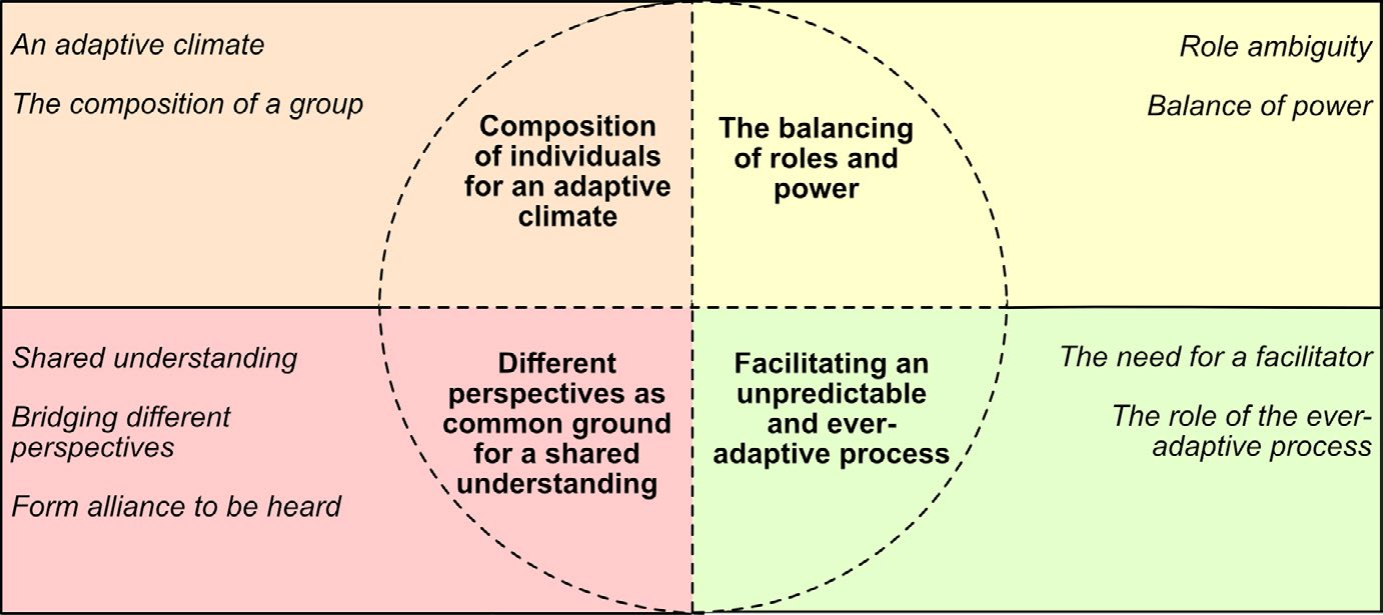
Subcategories and the associated categories in the results

The results showed how user participation manifested itself including potential enablers or barriers within a co‐design process involving patients, significant others and health‐care professionals. Four categories: ‘Composition of individuals for an adaptive climate’; ‘The balancing of roles and power’; ‘Different perspectives as common ground for a shared understanding’; and ‘Facilitating an unpredictable and ever‐adaptive process’, with all together nine subcategories, resulted from the analysis, illustrated in Figure 2.

### 3.1 Composition of individuals for an adaptive climate

#### 3.1.1 An adaptive climate

An adaptive approach to the needs of the fellow participants enabled participation. This included an adaptive climate to participants with different levels of communication skills through adapted conversation pace, allowing time to finish speaking and explanations to understand the task. This permissive, inclusive climate and collective engagement where the participants could express their opinion was facilitated by the participants’ responsiveness to each other, that is paid attention to, involved and encouraged each other to participate in the discussion and speak their minds. This was done by attentive and active listening with probes and encouragement, and affirmative body language.

A relaxed and easy‐going atmosphere where the participants spoke their minds enabled the forming of relationships with the other participants. This led to participants feeling comfortable in interacting with the other members of the group.

I think it’s important to be open‐minded – How should I put it? – that one is open. Everyone seemed to be very open hearted in all regards. Of course, I also opened up and they opened up as well. Interview, patient 1.

#### 3.1.2 The composition of a group

During the course of the workshops, it became evident that individual factors such as creativity skills, communication skills, competencies and ability to act within a group affected participation. Thus, the composition of individuals, rather than belonging to a profession or health‐care context, enabled and hindered participation within the groups.

Actually, I thought the groups were very good and then, if you want to make some kind of analysis of personalities, people do have different strengths. Some like to analyse, synthesise and formulate. Some like to create new ideas, be creative, play. Some like to observe. So, to create a really good team it is naturally quite appropriate to have people with different skills. Interview, facilitator, F1.

### 3.2 The balancing of roles and power

#### 3.2.1 Role ambiguity

Patients and the significant other struggled to combine their binary roles of being a ‘user’ with experience in the stroke care trajectory and being an equal, accountable partner in the co‐design group. One patient and the significant other requested more information about the health‐care context as they expressed that limited knowledge hindered their ability to contribute beyond their own experiences.

There was a clear clash of different identities at times; I’m not 100% certain that it works to have patients and family members as both team members and users – in the same project, on the same team. Interview, facilitator, F1.

The participants expressed ambiguity in what role they were expected to take in the design process. The ambiguity comprised several aspects: the roles within each group, the participants’ own view of their role, the binary roles of patients and the significant other as both users and members of the co‐design group, the professionals’ ambiguity of being users, professionals and a member of the co‐design group, and the traditional hierarchical health‐care roles as expert and novice. This meant insecurity in how to participate in the workshop activities.

I haven’t felt like I’ve been on equal terms as I haven’t known what you want me to do – should I help the patient as a member of staff or should I focus on my task as a participant Interview, health‐care professional, H5.

The collision between patients’ and the significant other's bad experience of the health‐care trajectory and the professional identity as provider of good quality health care was observed to hinder the adoption of equivalent roles. Some of the professionals struggled to resign from the role as professional, becoming defensive and questioning the experience of the patients/significant other.

There may be a general problem that emerges quite clearly in the group at times. There is a kind of accusation that the patients make due to painful, bad experiences they had of healthcare. Obviously, this clashes a great deal with your professional identity. It demands a great deal, to understand and not defend the system. It’s very difficult to separate the system you’ve worked for, and in, for so many years from yourself. Recorded reflection, workshop 4.

#### 3.2.2 Balance of power

There were examples when the view of participation was hierarchical and an uneven balance of power became evident. Some patients viewed the professionals as experts and felt that they as patients had less to contribute than the professionals who had everyday health‐care experience and skills.

I felt inferior in that way; of course, they were experts and I wasn’t. Interview, patient 2.

Health‐care professionals, in their turn, could steer the discourse by contrasting the experience‐based perspective of the patients/significant other from one unique occasion with their long‐term experience from working with these issues every day. This appeared to risk cementing the traditional expert‐novice roles. The use of role‐play, where participants were required to clarify and change roles, was experienced as neutralizing the roles between the patients/significant other and the professionals.

### 3.3 Different perspectives as common ground for a shared understanding

#### 3.3.1 Shared understanding

A shared understanding among the participants regarding the purpose and aim of the process appeared to enable equal participation. However, the pre‐understanding of what the co‐design process constituted and what it meant differed among the participants.

I think that it has worked well between us healthcare staff, and even with one of the family members. It was more difficult to work with the patient in our group, who found it difficult to understand the purpose of the workshops. Questionnaire, health‐care professional, H2.

Further, a continuous consensus and understanding between the participants within each group regarding topics of conversation, needs, insights, solutions and how to jointly execute and accomplish tasks enabled the participants’ possibility to participate on equal terms. Lack of shared understanding gave rise to frustration, which hindered the workflow, and in some cases even led to the exclusion of the person having difficulties understanding.

This pressure to come up with something – like, what you’re thinking about and writing it down. ‘What do you think about this in three words’. Your heart races. I can’t do this. I’m not in the swing of this and it might be difficult for us as family members who might not have the training either. Interview, significant other.

A recurrent use of cumbersome language, that is sector‐specific words from the design area including a mix of Swedish and English, hindered the understanding of new tasks and concepts used in the process. This, in turn, hindered the participants’ ability to comprehend the tasks that the groups would perform, limiting the possibility to participate as an equal group member.

What I found hard to understand was the process itself that she had drawn up and discussed. In some way it was above, or outside what I think is important. It was more fancy words than genuine. It was a little bit too flashy for my taste. Interview, significant other.

The use of paraphrasing and reflection, that is recapitulating what had been said and whether the interpretation was in line with the perception among the members of the group, helped to confirm a shared understanding among the participants and provided an opportunity to reach consensus. This was often practised by health‐care professionals. A shared understanding enabled ownership of the co‐design process and increased the motivation to participate wholeheartedly in the development of the new care transition.

#### 3.3.2 Bridging different perspectives

Participants described difficulties in bridging the experience‐based perspective of patients and the significant other and the perspective of the health‐care professionals. Even though they acknowledged the different perspectives as a strength, which enriched and broadened their understanding and view of the care trajectory and each other, it also posed some difficulties. As the co‐design process gave precedence to the needs of the patients and significant other, the health‐care professionals experienced that their perspective had to stand back. The perception of the actual needs of the patients and significant others differed, and sometimes, the participants had difficulties understanding each other's perspectives. This led to irritation and dissatisfaction within the group, difficulties in participating and, in some cases, also a distance between participants.

Well, patient and family members were more difficult to work with. As healthcare professionals, we think alike and know how things are done when we work, while patients/family members would like us to solve their particular problem. And it became, sort of the focus in the group that they talked about their needs, while we naturally see that, for you this might have been a good solution but for someone else it might not have been a good solution – perhaps we see the bigger picture while they might focus a bit more on their own experience. Interview, health‐care professional, H1.

In contrast, there were also examples of health‐care professionals who stated that the health‐care system´s perspective must be set aside in favour of the patients’/significant other's perspective and that the discussion must be based on the needs of the patients/ significant other.

In that respect I might not have based it an enormous amount on my own profession; rather, more from reasoning about what might be good from the patient’s perspective, and then perhaps my profession was not the most crucial aspect – it was more about getting inside the patient’s head. What would I have thought If I ended up in that situation? Interview, health‐care professional, H10.

#### 3.3.3 Form alliance to be heard

The formation of alliances was made by both patients/significant others and health‐care professionals and appeared to enhance the voice and perspective of the individual. The health‐care professionals’ alliances emanated from their clinical setting and the patients’ from their experience along the care trajectory. Participants could make their voices heard by the sharing and recognition of their experience in their respective alliance.

There was a certain tendency for the patient and neuroteam [multidisciplinary team] representative in the group to find symbioses in experiences and be able to reinforce one another’s narratives, while at the same time the speech and language therapist from the stroke unit and the occupational therapist from the geriatric stroke ward could find common ground based on the perspective from hospital care. Field notes, workshop 1.

### 3.4 Facilitating an unpredictable and ever‐adaptive process

#### 3.4.1 The need for a facilitator

The facilitator was an enabler for participation and the workflow of the process. The facilitator identified imbalances in participation and made sure that the perspective of the patients and significant others was highlighted. However, the facilitator encountered difficulties providing continuous guidance and intense facilitation to meet the needs of all three groups. Participation within the groups was hindered by the absence of a facilitator in each group, responsible for initiating and stimulating discussions and interactions. There was often hesitation around how to initiate new assignments, which caused worry and stress among the participants. As a response, an informal facilitator role emerged. Most of the time, the responsibility and informal facilitation ended up with one of the health‐care professionals within the groups, often the same person from time to time.

What one might have wished for was a little more facilitation of the groups, that someone had been present who could actually offer the groups further support, because they come up with good ideas and thoughts, but it feels like they need affirmation from someone. Recorded reflection, workshop 4.

#### 3.4.2 The role of the ever‐adaptive process

The co‐design process both seemed to enable and hinder participation. The diversity of assignments and methods achieved an open and creative environment and enabled a variety of ways for the participants to express themselves. The varied arrangement allowed for several modes of expression and met the needs of different individuals.

But it was good that there were different methods. There were lectures, we could discuss, we could paint, we could write; I thought that all of that kind of thing was great. Then perhaps one might start to think in a different way as well. It’s also good in as much as we are different people in the room and there is always something that suits someone. Interview, health‐care professional, H1.

On the other hand, parts of the co‐design process also seemed to negatively affect the participants’ possibilities to participate on equal terms. Assignments that were forced, unclear and difficult to understand hindered participation. The confusion that arose when the participants did not understand how to take on an assignment made participants cautious about speaking their minds and participating in the task. This insecurity, together with stress due to the time‐limited process, made the participants focus less on interactions between each other and more on the fulfilling of the given task. Time was mentioned as both an enabler to avoid getting stuck in endless discussions and a barrier to participation.

It was slightly stressful at times, bang, bang. So we had to finish some things when we had barely started. I could lose a little focus at times. Interview, patient 1.

As dissatisfaction, minor conflicts and co‐operation problems occurred along the process, the possibility to adapt and revise the process was necessary to enable participation. Customized support, such as individual meetings and simplified information between workshops, was provided. One patient desired to explain their own experiences in greater detail, and arrangements to meet these needs were provided. An ever‐adaptive process was dependent on a reflective approach from the researchers and facilitator to perceive the needs of the participants and pay attention to changes needed to be made.

## 4 DISCUSSION

This study described how user participation manifests itself including enablers or barriers within a co‐design process involving patients with stroke, a significant other and health‐care professionals. Four categories were identified; all included both enablers and barriers for participation: ‘Composition of individuals for an adaptive climate’; ‘The balancing of roles and power’; ‘Different perspectives as common ground for a shared understanding’; and ‘Facilitating an unpredictable and ever‐adaptive process’. Our results suggest that the individual's participation in a co‐design process manifests itself through the interaction of the group members and their skills to handle different perspectives, roles and assignments. The individuals’ participation is enabled by an ever‐adaptive facilitation of the co‐design process.

The establishment of a positive group climate and inclusive atmosphere was described as important aspects to enable trust, facilitate speaking out and participate in the co‐design process. These results are in line with previous studies^21^, ^30^, ^31^, ^32^ and also align with the first step of our co‐design process, which was to establish a mutual understanding, empathy and trust among the participants.^33^ Building relationships and creating social connections were experienced as important means to reach an inclusive climate within the group. However, even though diversity among participants is an important element for a creative co‐design and design thinking,^26^ this entailed difficulties and challenges in collaboration in practice. Factors such as personality, creativity and communication skills, and ability to act within a group affected how the participants interacted, collaborated and contributed to the process. The composition of groups with a majority of professionals and one single patient may also have affected the interaction. Putting together, participants in co‐design processes require careful consideration and the knowledge that a diversity of skills and capacities can require extensive efforts in facilitating collaboration within the groups.^34^ Our results point to the need for intense and adaptive facilitation to enable participation of all group members. Further, the necessity of teamwork in design education, to prepare designers/facilitators as they will be confronted with this matter in practice, has also been described.^35^ Co‐design includes a team approach,^36^ which stresses the importance of creating well‐working groups. Groups act and form collaboration in different ways depending on the composition of individuals.^37^ Groups who coordinate their work and manage group conflicts more easily create cohesiveness and an environment within the group where all members can contribute with their unique knowledge and skills.^37^, ^38^ This study highlights that to enable the participation of all participants, considerations must be taken concerning group composition and to support an inclusive group climate.

Ambiguity around the roles of the participants was described to hinder participation. Patients and significant others struggled to combine their binary roles as both users with lived experience and being an accountable partner in the design group. They experienced difficulties with contributing more than their own experience and felt they had insufficient knowledge (ie about the health‐care system) to participate and contribute to the discussions. Patients’ and the significant other's insecurity about their own ability to contribute^19^, ^21^ and difficulties in contributing more than their own experience have previously been reported.^31^ One of the main purposes of co‐design is to empower patients to become legitimate and acknowledged members of the design group.^37^ However, not all people, whether a patient, a significant other or a professional, are capable of assuming or want to assume the role of a designer in co‐producing health care.^3^ It has been recognized that the capability of individuals to participate in the co‐design varies^17^ and that users cannot always take on binary roles, as it depends on the level of expertise, passion and creativity of the user.^36^ In the present study, participation varied between participants and between different groups due to both capacities, will and contextual circumstances. Fischer points to the fact that people in some situations want to be designers and in other situations want to be ‘consumers’, and therefore advocates for the emergence of an ‘adaptive design’.^39^ This participation continuum has also been highlighted by Carman, showing that participation may vary depending on topic and context.^4^ This, together with the results of our study, highlights a need to allow for flexible levels of participation. Education of professionals and patients, to gain knowledge and skills about co‐design and the aim of the co‐design process, was requested and has been described to enhance the co‐design of new health‐care services^3^ and to strengthen their position within co‐design processes.^3^, ^16^ Our findings therefore call for specification and clarification of the different roles needed before recruiting to a co‐design process. This should also be clearly communicated to eligible participants. Further, it must be ensured that each of the participants understands their importance and unique significant value to the process.

The ambiguity of roles was also related to the power relationships between the participants which hindered participation. Some of the traditional hierarchical roles from health‐care practice were present in the co‐design workshops. The patients viewed the professionals as experts and downgraded their own experience as an equivalent contributor to the process, and some professionals considered patients’ experiences as anecdotal. This strengthened the boundaries between participants and hindered participation. This type of discreditation has been described by Fricker as epistemic injustice.^40^ Despite the fact that the co‐design process itself should facilitate a user‐centred approach with an emphasis on the experience of all users, it is of critical importance to acknowledge the possible presence of epistemic injustice. Hence, such injustice increases power asymmetries and separates rather than unites individuals. In the co‐design groups, an epistemic injustice could therefore be seen in how professionals valued their own and their peers’ long‐term experience more than the patients’/significant other's experience. However, there may also have been an epistemic injustice in that the workshops were based on the patients’/significant other's needs and their experiences were prioritized rather than the professionals’ work‐related experience. Since people's experiences are the focus of participatory design methods, all experiences should be recognized as a form of expert knowledge and valued equally as any type of knowledge.^41^ However, experience is often determined by tacit knowledge, which is typically hard to define, formalize and validate,^42^ requiring regular personal contact, dialogue and trust between people.^19^, ^24^ Despite our intention to have a user‐centred focus, and even though different techniques were used to create empathy, trust and mutual understanding,^26^ we did not fully succeed in an equal view on the value of experience. One way to overcome this obstacle might have been to deliberately address what experience is and what kind of experience was in focus in an open and reflective dialogue as part of the co‐design process.^19^, ^22^ One reason for the ambiguity of roles could have been unclear introduction of what was expected of the participants. To overcome this, more time and attention should have been provided to assure a shared understanding of the co‐design process including the moral and methodological underpinnings of user participation.^43^ Furthermore, the roles, responsibilities and objectives should have been more clearly defined to facilitate partnership and engagement.^16^, ^44^, ^45^ We suggest that the ever‐adaptive facilitation must consider the differences in the individuals, how used they are to work in co‐design processes and how much collaboration with each other the co‐design process entails.

The data collection with four different data sources generated rich data and enabled us to validate data through triangulation of sources, which strengthens the credibility of our study. Further, the use of both questionnaires and interviews enabled that participants in questionnaires to more freely express potential negative experiences of the process, while also ensuring rich data from the interviews. At the same time, conducting interviews with all the participants might have generated even richer data. The observers and interviewers were the same individuals who conducted the analysis, that is the researchers responsible for the project. This might have affected both the responses from the interviewees and the interpretation of the data. This in‐depth knowledge of the project and the data may have not only led to preconceptions but also enabled a rich understanding of the process. A reflexive approach was used by the researchers during the steps of data collection and analysis to acknowledge potential preconceived assumptions. The underrepresentation of patients and significant others might have affected the outcome of our results. However, involving health‐care professionals with different professions and from both hospital and primary care, together with patients and significant others, and at the same time facilitating participation for all involved stakeholders is difficult. We divided the participants into three groups to balance the representation and designed the co‐design process to facilitate participation. Despite this, patients and significant others were underrepresented in number in all groups. We do further acknowledge the lack of participation from users in the analysis and manufacturing of this manuscript.

Our study shows that participation varied between individuals, groups and steps within the process, and on the topic of discussions and motivation to contribute. It has been argued that a co‐design process inherently aims at collective ownership,^24^ that is the higher rungs on Arnstein's ladder of citizen participation.^1^ However, based on the result of our study, participation is not something that is realized by only applying participatory design methodology. Several authors have engaged in further elaboration on Arnstein's view of participation,^46^, ^47^, ^48^ Tritter and McCallum claim that Arnstein does not acknowledge the complexity, and dynamic and evolutionary nature of user participation.^46^ This is in line with how participation manifested itself in this study. Andersen et al. reason that participation should be a ‘matter of concern’ rather than a ‘matter of fact’.^48^ We acknowledge that the aim of co‐design processes might not be to strive towards the highest rung on Arnstein's participation ladder in terms of all participants involved, but that a coherent use of co‐design in the development of services might contribute to citizen power. Participation must be recognized and assessed on an individual basis and be voluntary in the sense that participants must have the possibility to refrain from participation and disclaim their right to have a say in all specific topics.

In conclusion, the individual's participation in a co‐design process manifests itself through the interaction of the group members and their skills to handle different perspectives, roles and assignments. The individuals’ participation is enabled by an ever‐adaptive facilitation. Furthermore, co‐design processes should allow for varying levels of participation among the group members and throughout the process. Future research should explore how different levels of individuals’ participation impact the outcome of the co‐design process.

## CONFLICT OF INTEREST

The authors declare no conflicts of interest.

## AUTHOR CONTRIBUTIONS

All authors of the manuscript have approved and agreed with its submission to the journal Health Expectations and have made significant contributions to the composition of the manuscript and meet the criteria for authorship. Sebastian Lindblom, Maria Flink, Marie Elf, Ann Charlotte Laska, Lena von Koch and Charlotte Ytterberg contributed to the study concept and design. Sebastian Lindblom, Maria Flink, Lena von Koch and Charlotte Ytterberg performed acquisition of data. Sebastian Lindblom, Maria Flink, Marie Elf, Ann Charlotte Laska, Lena von Koch and Charlotte Ytterberg analysed and interpreted the data. Sebastian Lindblom, Maria Flink, Lena von Koch and Charlotte Ytterberg drafted the manuscript. Sebastian Lindblom, Maria Flink, Marie Elf, Ann Charlotte Laska, Lena von Koch and Charlotte Ytterberg critically revised the manuscript for intellectual content.

## Data Availability

Research data are not shared due to privacy or ethical restrictions.

## APPENDIX 1

### INTERVIEW GUIDE

How did you experience participation in these workshops?; How do you think collaboration has worked with your group?; How did you experience the possibility to put forward what was important to you?; To what extent do you think you have been listened to?; In what way was it difficult/easy to put forward what you wanted to say?; How do you think the arrangement and content of the process have affected your ability to express your views?; How has it been for you to understand the perspective of the others?; What facilitated or hindered everyone's perspective from emerging?; How do you think the arrangement could have been made differently to enable everyone's participation on equal terms?

Probes and follow‐up questions were used to get more details of the views of the participants.

## References

[1] Arnstein SR . A ladder of citizen participation. J Am Inst Planners. 1969;35(4):216‐224.

[2] Institute of Medicine, Committee on Quality of Health Care in America . Crossing the Quality Chasm: A New Health System for the 21st Century. Washington, DC: National Academy Press; 2001.

[3] Batalden M , Batalden P , Margolis P , et al. Coproduction of healthcare service. BMJ Qual Safety. 2016;25(7):509‐517. 10.1136/bmjqs-2015-004315 PMC4941163 26376674

[4] Carman KL , Dardess P , Maurer M , et al. Patient and family engagement: a framework for understanding the elements and developing interventions and policies. Health Aff. 2013;32(2):223‐231. 10.1377/hlthaff.2012.1133 23381514

[5] Coulter A , Ellins J . Effectiveness of strategies for informing, educating, and involving patients. BMJ. 2007;335(7609):24‐27. 17615222 10.1136/bmj.39246.581169.80 PMC1910640

[6] Ekman I , Swedberg K , Taft C , et al. Person‐centered care–ready for prime time. Eur J Cardiovasc Nurs. 2011;10(4):248‐251. 21764386 10.1016/j.ejcnurse.2011.06.008

[7] Docteur E , Coulter A . (Highlight Report) Patient‐Centeredness in Sweden's Health System ‐‐ An External Assessment and Six Steps for Progress. The Swedish Agency for Health and Care Services (Vårdanalys); 2012;3. https://papers.ssrn.com/sol3/papers.cfm?abstract_id=2306631.

[8] Björgvinsson E , Ehn P , Hillgren P‐A . Participatory design and “democratizing innovation”. Proceedings of the 11th Biennial Participatory Design Conference, Sydney, Australia. 2010.

[9] Bate P , Robert G . Experience‐based design: from redesigning the system around the patient to co‐designing services with the patient. Qual Safety Health Care. 2006;15(5):307‐310. 10.1136/qshc.2005.016527 PMC2565809 17074863

[10] Blackwell RWN , Lowton K , Robert G , Grudzen C , Grocott P . Using experience‐based co‐design with older patients, their families and staff to improve palliative care experiences in the emergency department: A reflective critique on the process and outcomes. Int J Nurs Studies. 2017;68:83‐94. 10.1016/j.ijnurstu.2017.01.002 28095347

[11] Hoeeg D , Christensen U , Grabowski D . Co‐designing an intervention to prevent overweight and obesity among young children and their families in a disadvantaged municipality: methodological barriers and potentials. Int J Environ Res Public Health. 2019;16(24):15. 10.3390/ijerph16245110 PMC6950007 31847342

[12] Thinyane M , Bhat K , Goldkind L , Cannanure VK . The messy complexities of democratic engagement and empowerment in participatory design ‐ an illustrative case with a community‐based organisation. CoDesign. 2020;16(1):29‐44.

[13] Palmer VJ , Weavell W , Callander R , et al. The participatory zeitgeist: an explanatory theoretical model of change in an era of coproduction and codesign in healthcare improvement. Med Humanit. 2019;45:247‐257. 29954854 10.1136/medhum-2017-011398 PMC6818522

[14] Brett J , Staniszewska S , Mockford C , et al. A systematic review of the impact of patient and public involvement on service users, researchers and communities. The Patient. 2014;7(4):387‐395. 25034612 10.1007/s40271-014-0065-0

[15] Voorberg WH , Bekkers VJJM , Tummers LG . A systematic review of co‐creation and co‐production: embarking on the social innovation journey. Public Manag Rev. 2015;17(9):1333‐1357.

[16] Bombard Y , Baker GR , Orlando E , et al. Engaging patients to improve quality of care: a systematic review. Implement Sci. 2018;13(1):98. 30045735 10.1186/s13012-018-0784-z PMC6060529

[17] Holland‐Hart DM , Addis SM , Edwards A , Kenkre JE , Wood F . Coproduction and health: Public and clinicians' perceptions of the barriers and facilitators. Health Expect. 2019;22(1):93‐101. 30289592 10.1111/hex.12834 PMC6351407

[18] Ocloo J , Matthews R . From tokenism to empowerment: progressing patient and public involvement in healthcare improvement. BMJ Qual Safety. 2016;25(8):626‐632. 10.1136/bmjqs-2015-004839 PMC4975844 26993640

[19] Gustavsson SMK , Andersson T . Patient involvement 2.0: Experience‐based co‐design supported by action research. Act Res. 2019;17(4):469‐491.

[20] Revenäs Å , Hvitfeldt Forsberg H , Granström E , Wannheden C . Co‐designing an eHealth service for the co‐care of Parkinson disease: explorative study of values and challenges. JMIR Res Protocol. 2018;7(10):e11278. 10.2196/11278 PMC6234336 30377143

[21] Pallesen KS , Rogers L , Anjara S , De Brún A , McAuliffe E . A qualitative evaluation of participants' experiences of using co‐design to develop a collective leadership educational intervention for health‐care teams. Health Expect. 2020;23(2):358‐367. 31999883 10.1111/hex.13002 PMC7104638

[22] Farr M . Power dynamics and collaborative mechanisms in co‐production and co‐design processes. Critical Social Policy. 2017;38(4):623‐644.

[23] Palmer VJ . The participatory zeitgeist in health care: it is time for a science of participation. J Participatory Med. 2020;12(1):e15101. 10.2196/15101 PMC7434075 33064092

[24] Donetto S , Pierri P , Tsianakas V , Robert G . Experience‐based co‐design and healthcare improvement: realizing participatory design in the public sector. Design J. 2015;18(2):227‐248.

[25] Bjögvinsson E , Ehn P , Hillgren P‐A . Design things and design thinking: contemporary participatory design challenges. Design Issues. 2012;28(3):101‐116.

[26] Carlgren L , Rauth I , Elmquist M . Framing design thinking: the concept in idea and enactment. Creat Innovat Manag. 2016;25(1):38‐57.

[27] Lindblom S , Ytterberg C , Elf M , Flink M . Perceptive dialogue for linking stakeholders and units during care transitions ‐ a qualitative study of people with stroke, significant others and healthcare professionals in Sweden. Int J Integr Care. 2020;20(1):11. 10.5334/ijic.4689 PMC7101013 32256255

[28] Council BD . The design process: what is the double diamond. https://www.designcouncil.org.uk/news‐opinion/what‐framework‐innovation‐design‐councils‐evolved‐double‐diamond. Accessed 15 April 2020.

[29] Graneheim UH , Lundman B . Qualitative content analysis in nursing research: concepts, procedures and measures to achieve trustworthiness. Nurse Educ Today. 2004;24(2):105‐112. 14769454 10.1016/j.nedt.2003.10.001

[30] Howe A , Mathie E , Munday D , et al. Learning to work together ‐ lessons from a reflective analysis of a research project on public involvement. Res Involv Engag. 2017;3:1. 10.1186/s40900-016-0051-x PMC5611599 29062526

[31] Haines KJ , Holdsworth C , Cranwell K , et al. Development of a peer support model using experience‐based co‐design to improve critical care recovery. Crit Care Explor. 2019;1(3):e0006. 32166251 10.1097/CCE.0000000000000006 PMC7063862

[32] Harrison M , Palmer R . Exploring patient and public involvement in stroke research: a qualitative study. Disabil Rehabil. 2015;37(23):2174‐2183. 25598139 10.3109/09638288.2014.1001525

[33] Gasparini A . Perspective and use of empathy in design thinking. Paper presented at: ACHI, the eight international conference on advances in computer‐human interactions, 2015.

[34] Trischler J , Kristensson P , Scott D . Team diversity and its management in a co‐design team. J Serv Manag. 2018;29(1):120‐145.

[35] Stempfle J , Badke‐Schaub P . Thinking in design teams ‐ an analysis of team communication. Des Stud. 2002;23(5):473‐496.

[36] Sanders EBN , Stappers PJ . Co‐creation and the new landscapes of design. CoDesign. 2008;4(1):5‐18.

[37] Trischler J , Pervan SJ , Kelly SJ , Scott DR . The value of codesign: the effect of customer involvement in service design teams. J Serv Res. 2018;21(1):75‐100.

[38] DeChurch LA , Mesmer‐Magnus JR , Doty D . Moving beyond relationship and task conflict: toward a process‐state perspective. J Appl Psychol. 2013;98(4):559‐578. 23731027 10.1037/a0032896

[39] Fischer G . Beyond "Couch Potatoes": from consumers to designers and active contributors. First Monday. 2002;7 (12):10.5210/fm.v7i12.1010.

[40] Fricker M . Epistemic Injustice: Power and the Ethics of Knowing. Oxford: Oxford University Press; 2007.

[41] Tambuyzer E , Pieters G , Van Audenhove C . Patient involvement in mental health care: one size does not fit all. Health Expect. 2014;17(1):138‐150. 22070468 10.1111/j.1369-7625.2011.00743.x PMC5060706

[42] Spinuzzi C . The methodology of participatory design. Tech Commun. 2005;52(2):163‐174.

[43] Wilson P , Mathie E , Keenan J , et al. Research with patient and public involvement: a realist evaluation – the RAPPORT study. Health Services and Delivery Research. 2015;3(38):1–176. 10.3310/hsdr03380. 26378332

[44] Brown LJE , Dickinson T , Smith S , et al. Openness, inclusion and transparency in the practice of public involvement in research: A reflective exercise to develop best practice recommendations. Health Expect. 2018;21(2):441‐447. 29105227 10.1111/hex.12609 PMC5867325

[45] Bird M , Ouellette C , Whitmore C , et al. Preparing for patient partnership: A scoping review of patient partner engagement and evaluation in research. Health Expect. 2020;23(3):523‐539. 32157777 10.1111/hex.13040 PMC7321722

[46] Tritter JQ , McCallum A . The snakes and ladders of user involvement: Moving beyond Arnstein. Health Policy. 2006;76(2):156‐168. 16006004 10.1016/j.healthpol.2005.05.008

[47] Halskov K , Hansen NB . The diversity of participatory design research practice at PDC 2002–2012. Int J Hum Comput Stud. 2015;74:81‐92.

[48] Andersen LB , Danholt P , Halskov K , Hansen NB , Lauritsen P . Participation as a matter of concern in participatory design. CoDesign. 2015;11(3–4):250‐261.

[49] Duncan PW , Bode RK , Min Lai S , Perera S . Rasch analysis of a new stroke‐specific outcome scale: the Stroke Impact Scale. Arch Phys Med Rehabil. 2003;84(7):950‐963. 12881816 10.1016/s0003-9993(03)00035-2

